# Sustainable leadership: A literature review and prospects for future research

**DOI:** 10.3389/fpsyg.2022.1045570

**Published:** 2022-11-07

**Authors:** Yaohua Liao

**Affiliations:** Modern School of Management, Zhejiang Industry and Trade Vocational College, Wenzhou, China

**Keywords:** sustainable leadership, sustainability, economic benefits, social responsibility, environmental protection

## Abstract

The various social issues that accompany economic development pose new challenges for leaders to integrate economic benefits, social responsibility, and environmental protection. In this context, various new leadership styles have emerged. Among them, sustainable leadership reveals the key role of leaders in balancing the triple goals of economy, society and environment, and has become an important part of leadership theory research in recent years. We searched the literature related to sustainable leadership in databases such as Web of Science, EBSCO and CNKI. Based on the existing literature, we systematically review the origins, connotations, analytical perspectives, measurement methods, and conceptual comparisons of sustainable leadership. And we also construct an integrated analytical framework of sustainable leadership on the premise of sorting out and summarizing the antecedents and consequences of sustainable leadership. Finally, we provide an outlook on the future research areas of sustainable leadership in order to further promote research of sustainable leadership.

## Introduction

Whether philosophical thinking—“harmonious coexistence between man and nature” or environmental protection in the era of industrial civilization, the concept of sustainable development has always played an important role in guiding global actions (Piwowar-Sulej et al., 2021). In particular, the promulgation of “transforming our world: 2030 agenda for sustainable development” in 2015 has ushered in a new era of global sustainable development. Meanwhile, to strengthen human responsibility for sustainable development, in 2015, 193 ONU member states approved 17 Sustainable Development Goals (SDGs). These goals require countries, organizations and individuals to take action for sustainable development (Cesário et al., 2022). Sustainable development is a highly diverse and dynamic system (Dos Santos and Ahmad, 2020), which is not only aimed at solving the challenges related to climate change and environmental degradation, such as the pollution of air, water and soil, overfishing and species extinction but also related to health, wellbeing and the elimination of inequality and poverty. Due to the close relationship between the organizations and the sustainable development of social economy, some scholars pointed out that this goal can be achieved when enterprises fully accept the challenge of sustainable development, take it as an opportunity for business development, and change their business models (Tideman et al., 2013). At the same time, scholars from different disciplines agree that although enterprises are one of the key perpetrators of these problems, they also play an important role in solving these problems (Piwowar-Sulej et al., 2021). There is no doubt that enterprises have become the basic unit to ensure the implementation of sustainable development. But, for a long time, it has been difficult for enterprises to embed the concept of sustainable development into their own business operations and instead perform their social responsibilities in the form of charitable donations or public welfare activities, resulting in the concept of sustainable development becoming a mere form of social responsibility. However, it should be emphasized that the implementation of these activities of sustainable development is uncertainty. If the enterprise does not have high performance, it is impossible to give better consideration to sustainability (Suriyankietkaew and Avery, 2016). To solve this problem, scholars are actively seeking solutions, and find that introducing the concept of sustainable development into leadership is the key to change the situation and build a sustainable organization (Gerard et al., 2017). Based on this, sustainable leadership, which promotes enterprises to advance toward sustainable development, has attracted increasing attention (Dalati et al., 2017; Piwowar-Sulej et al., 2021). Sustainable leadership is the product of the integration of sustainable development and leadership, which is a kind of leadership and management behavior that aims to meet the needs of stakeholders and develop the core business of the enterprise, so as to create long-term value for all stakeholders. It goes beyond the green transformational leadership and responsible leadership, and emphasizes the balanced development of economy, society, and environment. At the same time, it is conducive to helping enterprises achieve profitable growth and sustainability, and has become one of the hotpots of current leadership research (Avery and Bergsteiner, 2011a,b).

The existing research on sustainable leadership has made progress, and some scholars have found that sustainable leadership have a positive impact on employees’ organizational commitment, employees’ job satisfaction (Suriyankietkaew and Avery, 2014), employees’ organizational trust (Dalati et al., 2017), organizational sustainable performance (Burawat, 2019; Iqbal et al., 2020a,b), organizational financial performance (Kantabutra and Thepha-Aphiraks, 2016; Suriyankietkaew and Avery, 2016) and organizational resilience (Avery and Bergsteiner, 2011b). However, the research of sustainable leadership is still in infancy, and there is a lack of systematic review in this field, which is in sharp contrast to the calls for sustainable leadership. In order to better promote the theoretical research and management practice of sustainable leadership, we searched the literature related to sustainable leadership in databases such as Web of Science, EBSCO and CNKI. We also sort out the existing literature on sustainable leadership, and systematically review, comb and comment on its origin, connotation, analytical perspective, antecedents and outcomes, construct an integrated research framework for sustainable leadership, and prospects for future research.

## The concept of sustainable leadership

### The origin of sustainable leadership

The concept of sustainable development into the field of organizational management was introduced by Brundtland committee. They pointed out that sustainable development is a way of development that meets current needs without harming future generations’ needs. Later, this concept has aroused intense discussion in the academic circles and is still under extensive research. For example, Pearce and Turner (1990) put forward the circular economy model, emphasizing the interdependence between economy and environment. Weale (1992) believed that sustainable development challenges the mutually exclusive relationship between economy and environment, which makes the conflict between them conceptualized again. On this basis, Elkington and Rowlands (1999) proposed a triple bottom line framework for sustainable development, indicating that social, environmental (ecological) and financial (economic) indicators are the balance methods to measure enterprise business performance. That is, enterprises should not only pay attention to the profit and loss account, but also consider their own impact on the environment and social responsibility, which means that enterprises need to reduce the negative impact of economic growth to enhance the continuity of development. At the same time, existing studies have shown that the process of seeking sustainable development for enterprises that can carry out sustainable entrepreneurship has also won new opportunities for them (Kumar and Kiran, 2017).

However, with the increasing social and environmental problems arising from economic development, there is a serious imbalance between economic benefits, social responsibility, and environmental protection in the process of enterprise operation. How to overcome this imbalance and achieve the goals of improving performance, resilience and sustainability has become a focus topic of common concern in the practical and theoretical circles. In order to better take into account a wider range of stakeholders, leaders need to establish the concept of sustainable development, embed it into the organization, and implement sustainable leadership behavior as the leader is the key to the enterprise transformation (Avery, 2005). Hargreaves and Fink (2004) and Avery (2005) combined the concept of sustainable development with leadership and put forward the concept of sustainable leadership. Since then, many scholars have explored its connotation based on different context.

### The connotation of sustainable leadership

Hargreaves and Fink (2004) developed a model of sustainable leadership based on the educational organization, and proposed that sustainable leadership in education refers to the ability to maintain and promote in-depth and extensive learning (depth); make plans and preparations for succession in order to ensure long-term development (sustainability); emphasize dialogue, common development and shared decision-making (breadth); actively share knowledge and resources with neighboring schools or communities to improve the environment (justice); avoid consistency and standardization of policies, curricula, assessments and training to promote diversity (diversity); provide incentive and reward policies to attract talents, and establish a network to enhance mutual learning and support (resourcefulness); respect the past experience of leadership behavior and learn from it in pursuit of creating a better future (maintenance).

With the deepening of theoretical research, Avery (2005) introduced the concept of sustainable leadership into the field of enterprise management for the first time, and proposed a new concept of sustainable leadership based on comparing the differential impact of the two development models of capitalism—the British American model and the Rhine model on the leadership style of organizational managers, and summarized 19 elements. Avery (2005) pointed out that sustainable leadership means having long-term decision-making ability, promoting systematic innovation, cultivating a loyal staff team, and providing high-quality products, services and solutions. Its purpose is to balance the relationship between people, profits and the earth, and promote the sustainability of the enterprise through corresponding management practices. These management practices cover management systems, principles, processes and values, and can form a self-reinforcing leadership system within the organization, involving CEO role change, decentralized decision-making, ethical behavior, high social responsibility and high environmental responsibility. Based on this study, Avery and Bergsteiner (2011a,b) identified four additional practices (self-management, trust, innovation, and job involvement) and integrated them with the initial 19 elements to finally form a sustainable leadership framework including 23 elements. The framework is arranged in the form of a pyramid, reflecting the development concept of mutual support and interdependence. When lower level practices are in place, they will promote and support the emergence of higher-level practices, and higher-level practices in turn rely on the existence of these basic elements.

In general, the 23 practices of sustainable leadership are divided into three levels: basic practice, high-level practice and key performance drivers. Basic practice is at the bottom of the pyramid, including 14 basic practice activities, such as continuously developing every employee within the organization, seeking cooperative labor relations, long-term perspective and a wide range of stakeholder responsibilities; High level practice is at the second level of the pyramid, covering the creation of self-management employees, the use of team strength and knowledge sharing; The key performance drivers are the third layer of the pyramid, including innovation, emotional input and high quality, which essentially improve the customer experience and promote the development of organizational performance. Therefore, at the top of the pyramid is its possible performance results, such as brand and reputation, long-term value to multiple stakeholders, etc.

### The analytical perspective of sustainable leadership

In the process of the continuous development of the research by scholars such as Hargreaves and Fink (2004), other scholars followed them but put forward supplementary definitions or new definitions based on different situations (Lambert, 2012; Tideman et al., 2013; Gerard et al., 2017). Some scholars also focused on the individual characteristics and personal behaviors of sustainable leadership and analyzed its connotation (Casserley and Critchley, 2010). Others explained its concept from a cross-layer perspective (Armani et al., 2020). Based on these scholars’ researches of sustainable leadership, we analyze sustainable leadership from three levels and five perspectives, as shown in Table 1.

**TABLE 1:** The connotation and analytical perspective of sustainable leadership.

Level	Perspective	Key features	Specific performance
Individual level	Individual characteristic	Sustainable consciousness and values; How to cultivate sustainable consciousness and values	Moral values and principles, develop people; Action reflection (learning while doing), mental intelligence (clear goals, situational awareness), physical health (stress management, self-care); Environment shapes sustainable leaders
Organizational level	Organizational culture	Emphasize the importance of leadership to a sustainable organizational culture	Encourage a green, innovative and sustainable organizational culture; Cultivate a strong and widely shared organizational culture;
	Strategic orientation	Emphasize that leadership helps to promote the formation of sustainable development strategies of organizations	Promote leadership → enterprise strategic orientation → sustainable organization/performance Expand the value chain of strategic decision-making to the social environment
	Human resource development	Human resource development through Sustainable Leadership	Regard employees as one of the stakeholders of the enterprise to cultivate their ability to continuously develop themselves
Cross level	Interaction between individual and organization	Emphasize leadership integration and promote the correlation between multiple individual and organizational factors	Integrate personal practices with organizational initiatives Context, awareness, continuity, connection, creativity and collective leadership

The author collates according to relevant literature.

At the individual level, sustainable leadership mainly relies on the sustainable individual characteristics of leaders to create sustainable organizations. Sustainable leadership from the characteristic perspective refers to the values of sustainable development possessed by leaders and their sustainable consciousness embodied in their work. In organizations, some leaders are more likely to adopt a responsible attitude toward sustainable initiatives and activities, which largely depends on the leader’s personal characteristics (Waldman and Siegel, 2008). For example, Renwick et al. (2013) emphasized the importance of individual characteristics of leaders, such as moral values and principles, in implementing sustainable measures in enterprises. Casserley and Critchley (2010) pointed out that leaders’ attention to their own psychological and physical health needs is a prerequisite to ensure the effectiveness of their sustainable development leadership. On this basis, leaders would create a sustainable organizational environment, establish sustainable organizational goals and play a responsible role to protect the ecological environment. In addition, the level of sustainable development awareness of leaders also plays a key role in helping them cope with the complex economic, social and environmental situations, and make changes when necessary, so as to promote the sustainable innovation of organizations (Macke and Genari, 2019).

At the organizational level, sustainable leadership is understood as a leadership activity that is embedded in the whole organization and forms a self-reinforcing system within the organization through relevant practices, so that enterprises can maintain economic, social and environmental balance in the whole life cycle, while helping organizations achieve long-term sustainable development. The literature on sustainable leadership at the organizational level can be analyzed from three perspectives: organizational culture, strategic orientation and human resource development. First, from the perspective of organizational culture, sustainable leadership is the key promoter of an organizational culture that focuses on innovation and sustainability within an organization (Avery and Bergsteiner, 2011b). At the same time, this organizational culture is an important factor in shaping sustainable leadership (Amar, 2019). Therefore, under the relevant interaction and matching of them, the organization’s sustainable development goals can be achieved. For example, some scholars pointed out that senior leaders’ encouragement of a green, innovative and sustainable organizational culture is conducive to enabling employees to have the same environmental and social goals, thus having a positive impact on the sustainable development of the organization (McCann and Sweet, 2014). Focusing on the long term, “doing the right thing”, promoting sustainable shared value creation and innovating sustainable business models are all basic skills and practices of sustainable leadership, which are conducive to improving organizational culture (Tideman et al., 2013). In particular, Avery and Bergsteiner (2011b) clearly put forward that cultivating a strong and shared organizational culture is one of the high-level practices of sustainable leadership, and regarded it as a goal to be pursued in a longer time after anchoring basic elements. Second, from the perspective of strategic orientation. Sustainable leadership can not only consider the complex interrelationship between individuals, business communities, natural environment and market demand, but also expand it to the value chain of enterprises based on strategic decision-making to obtain long-term achievements, while caring about social welfare, and protecting ecosystems (Burawat, 2019). Third, from the perspective of human resource development. Organizations can implement human resource development through sustainable leadership. Sustainable leadership includes all aspects of people-oriented management, regards employees as one of the stakeholders of the enterprise, and then regards it as the responsibility to cultivate a loyal and highly engaged staff team (Avery and Bergsteiner, 2011b). Sustainable leaders attach importance to and develop employees, and in return, employees support leaders and can show sustainable behaviors similar to leaders (Iqbal et al., 2020b). Therefore, sustainable leadership not only enables employees to develop themselves, but also makes this development sustainable (Gilley et al., 2011). In addition, the encouragement and support of sustainable leadership also helps to promote the growth of human resource developers, making them more patient and caring for employees.

In terms of cross level, sustainable leadership emphasizes that on the basis of understanding the new paradigm of economics and business trends, leaders can integrate their sustainability vision into the development of the organization more easily by combining the organizational perspective with the individual perspective, so as to promote the transformation of the organization to sustainable business, and also contribute to the construction of a sustainable economic system (Armani et al., 2020). Relevant studies can be analyzed from the perspective of interaction between individuals and organizations. The concept of interaction between individual and organization mainly believes that sustainable leadership not only covers the individual characteristics, organizational culture and strategy of leaders, but also has some intersections that can integrate them. For example, Armani et al. (2020) pointed out that interpersonal skills and change direction belongs to the leader’s individual characteristics, focus on stakeholders, seek sustainability in strategic and specific business outlook is related to the organization, and pay attention to sustainability, and coordinated organization culture and moral values and principles in the intersection, can into the personal practice and organizational measures. Tideman et al. (2013) pointed out that sustainable leadership is a kind of leadership behavior generated from the current situation of leaders and organizations under the background of recognizing the disruptive and transformational changes in current business and society. Whether it can promote the sustainable business transformation within the organization depends on the interaction of leadership awareness and situation in time and space.

## Concept comparison and measurement of sustainable leadership

### Comparison between sustainable leadership and similar leadership styles

The proposal of sustainable leadership provides a way to interpret the concept of sustainable development from the perspective of leadership. In recent years, some scholars have compared sustainable leadership with other similar leadership styles (Tideman et al., 2013). Comparing it with transformational leadership, green transformational leadership, responsible leadership and moral leadership in terms of structural dimension, principle, mode of action and motivation is conducive to a better understanding of the connotation of sustainable leadership (shown in Table 2).

**TABLE 2:** The comparison of leadership style.

Leadership type	Construct dimension	Principle	Mode of action	Motivation
Green transformational leadership	Green personalized care, green intelligence stimulation, green charisma, green Charm	Instill green values and environmental goals into followers and motivate them to exceed the expected level of environmental performance	Rely on leaders’ Environmental Protection examples and the transmission of green values	Enhance the green innovation of the organization, stimulate the green creativity and green behavior of employees, and achieve excess environmental performance
Moral leadership	People oriented, moral consciousness (moral quality and behavior), the formulation of moral standards and principles, and transparent decision-making style, etc.	Code of ethics	Set an example and moral rewards and punishments for followers through the moral exemplary role of leaders	Formation of employees’ moral behavior
Responsible leadership	Effectiveness, ethics and sustainability	Normative principles	Democratic consultation and active dialogue with stakeholders	Promote the long-term development of the organization by focusing on social responsibility and business ethics
Sustainable Leadership	Focus on the situation, show moral courage and high self-awareness, long-term vision, meet the needs of stakeholders, create sustainable shared value, and collective influence	Continuous learning, long-term success, maintaining others, social justice, development, developing environmental diversity and actively participating in environmental protection	Rely on the interaction of leaders’ leadership consciousness and situational consciousness in time and space	Pursue the balance of economic, social and ecological goals while achieving high performance, resilience and sustainability

The author collated according to relevant literature.

#### Green transformational leadership

Transformational leaders mainly show four skills or talents: cultivating the internal motivation of their followers (personalized care), being good at taking risks and improving their creativity by stimulating their independent thinking ability (intellectual stimulation), conveying their vision and instilling a sense of purpose and significance (charisma), and setting an example of honesty, reliability and morality for their followers (charm) (Bass et al., 1987). And green transformational leadership is the behavior that can motivate followers to achieve environmental goals and encourage them to exceed the expected level of environmental performance (Chen and Chang, 2013). If a transformational leader happens to have green values and can enhance the green creativity and green behavior of his subordinates through personalized care, intellectual stimulation, vision transmission and charisma, then this transformational leadership behavior is green transformational leadership (Wang et al., 2018). Therefore, the essence of green transformational leadership is still transformational leadership, and its dimensions, principles, modes of action and influence motivation are still consistent with transformational leadership, but green value orientation is added on the basis of the four dimensions of transformational leadership (Taşçı and Titrek, 2019). Although the green transformational leadership brings into the transformational leadership behavior the consideration of environmental issues, the attention to the product life cycle and the social and environmental impact of the whole supply chain, its goal is limited to passing on the values of green environmental protection, promoting green innovation, green environmental protection behavior and green product development. The breadth and depth of its connotation are still lower than that of sustainable leadership.

#### Ethical leadership

Ethical leadership means that leaders can not only show ethical behavior within the organization, but also promote followers to form an ethical behavior through decision-making and the process of interaction and communication with followers (Brown et al., 2005). The composition of ethical leadership revolves around ethical norms, involving people-oriented, ethical consciousness (ethical quality and behavior), the formulation of ethical standards and principles, and transparent decision-making style. Its focus is still limited to the binary relationship between leaders and followers, aiming to set an example for followers through the ethical exemplary role of leaders, so as to form an ethical atmosphere within the organization, such as public-private distinction, integrity, kindness and integrity, respect and tolerance, and further affect the behavior of followers. Some scholars also pointed out that ethical leadership can also affect the behavior of followers through moral rewards and punishments. Sustainable leadership also practices ethical principles, but it emphasizes the moral principles centered on the environment and community, which goes beyond the scope of moral leadership. In addition, sustainable leadership means cooperation rather than leading others, so it generally does not affect followers through rewards and punishments (Brown et al., 2005).

#### Responsible leadership

Maak and Pless (2006) put forward the concept of responsible leadership in the research of social responsibility integration leadership, which refers to the ability to establish and maintain trust and common collaborative relationships with stakeholders, and pursue common business vision. Some scholars also understand responsible leadership as the behavior of leaders practicing social responsibility. Both sustainable leadership and responsible leadership extend the relationship between leaders and followers to stakeholders inside and outside the organization, and have similarities in paying attention to social responsibility. However, responsible leadership emphasizes that leaders can promote the development of the organization by paying attention to social responsibility and business ethics. The key of sustainable leadership is not only embodied in ethical, social and responsible business behavior, but also rooted in the triple bottom line of economy, society and environment. Its purpose is to create long-term wellbeing and lasting value for all stakeholders, not just social and environmental responsibility. Sustainable leadership seeks to maintain an appropriate balance between economy, society and ecology while achieving high performance, resilience and sustainability (Burawat, 2019), and goes beyond the concept of green and social responsibility in enterprises. Although responsible leadership has expanded its focus from the relationship between leaders and followers to stakeholders, it still takes the current situation of organizations as the starting point (Tideman et al., 2013). In addition, responsible leadership implements democratic consultation with stakeholders, while sustainable leadership transcends its own interests by playing a beneficial role in society, which in turn enables it to achieve performance growth, resilience and sustainability, thus ensuring the balance of economy, society and ecology.

### The measurement of sustainable leadership

The measurement of sustainable leadership is primarily found in two areas: education and business management. There are many researches within the field of education. In terms of qualitative analysis, based on a comparison of different approaches to sustainable and unsustainable leadership in schools, Hargreaves and Fink (2004) summarized sustainable leadership around sustainable learning, environmental protection, and social justice. Burns et al. (2015) examined sustainable leadership in terms of observation and self-awareness, reflection, exploration of ecological and diversity perspectives, and learning from experience and community. Later, Taşçı and Titrek (2019) delved into sustainable leadership affecting lifelong learning in education, observing and asking questions about organizational vision improvement, social responsibility implementation and ethical standard setting. In terms of quantitative research, Farooq and Ibrahim (2017) developed a 25-item sustainable leadership questionnaire with 4 dimensions (staff capacity building, diversity, maintenance, and strategic leadership allocation) through an exploratory factor analysis of 300 administrative and academic staff questionnaires from 6 universities, sample item: “My university provides training opportunities for staff in leadership development programs.” Çayak (2021) developed a 36-item questionnaire with 4 dimensions (administration, economy, culture, and social sustainability) to measure the level of sustainable leadership of principals, sample item: “My principal tells his teachers about his sustainability practices.” In addition, leadership behaviors that promote sustainability in schools have also been investigated through a combination of qualitative and quantitative methods, such as Lambert (2012) who developed a sustainable leadership framework for colleges of continuing education through interviews and questionnaires to collect data consisting of 6 factors, including developing staff capacity, strategy and partnership building, developing long-term goals from short-term goals, diversifying workplace and curriculum development.

Current research within the field of business management has mainly used quantitative analysis, such as Avery and Bergsteiner (2011a) who designed a set of sustainable leadership questionnaires including 57 measures based on 23 practices of sustainable leadership. Later, Suriyankietkaew and Avery (2014) confirmed the validity of this questionnaire with a sample of 1,152 employees in Thai SMEs. Dalati (2015) developed a 10-item sustainable leadership questionnaire, sample item: “I have a good understanding of leadership.” Lee (2017) measures sustainable leadership in 5 dimensions: cohesive diversity, organizational justice, employee development, advancement orientation and work-life balance, with each dimension measured by 2 questions, sample item: “My supervisor works well with employees from diverse backgrounds.” McCann and Holt (2010) developed and empirically tested a sustainable leadership questionnaire based on sustainable leadership thinking and the ten pillars of sustainable leadership (e.g., social and environmental awareness, adaptability, patience), including 15 questions, sample item: “My leader cares about how sustainability affects employees.” This questionnaire has since been widely used in many studies such as McCann and Sweet (2014), Al-Zawahreh et al. (2019), Iqbal et al. (2020b), and Javed et al. (2020). Although there are more questionnaires for measuring sustainable leadership, the 15-item questionnaire developed by McCann and Holt (2010) has been more recognized and applied by scholars.

## The antecedents and consequences of sustainable leadership

### The antecedents of sustainable leadership

Previous scholars have discussed less on the antecedents of sustainable leadership, and the relevant studies are mainly in two aspects: individual and organizational contextual factors. In terms of individual factors, Taşçı and Titrek (2019) and Armani et al. (2020) pointed out that developing managers’ self-awareness can enhance sustainable leadership, because the development of sustainability relied on the way managers view the world and the importance they placed on certain organizational behaviors that involved ethical issues. Cheng et al. (2021) pointed out that many individual characteristics, such as humility, cognition, and integrity, can positively influence sustainable leadership, but this promotion was more likely to occur in highly ethical organizations. In terms of organizational contextual factors, Shaaban (2020) discussed the concept of responsible leadership and sustainable leadership and empirically tested it with a sample of 250 employees and leaders from 18 companies in Egypt, confirming the facilitative effect of responsible leadership on sustainable leadership.

### The consequences of sustainable leadership

Although previous scholars have noted that the consequences of sustainable leadership can manifest at the individual, team, organizational, and societal levels, existing empirical analyses have focused primarily on the individual and organizational aspects.

#### Individual level

The impact of sustainable leadership on employees is mainly reflected in two aspects: cognition and behavior. On the cognitive side, Suriyankietkaew and Avery (2014) stated that 20 out of 23 practices of sustainable leadership can significantly improve employees’ job satisfaction. Similarly, Lee (2017) identified several sustainable leadership practices as important predictor variables of employee satisfaction, such as work-family balance. Çayak and Çetin (2018) examined the impact of school principals’ sustainable leadership behaviors on teachers’ organizational commitment and job satisfaction and found that sustainable leadership could predict high levels of teachers’ organizational commitment and job satisfaction. Dalati et al. (2017) also examined the influence of sustainable leadership with teachers and found that sustainable leadership can increase the level of trust of employees in the organization. In terms of behavior, Shaaban (2020) argued that sustainable leadership improved employees’ behavior thus making them responsible employees. Moreira et al. (2022) believed that sustainable leadership enables employees to feel that the organization cared about them and valued their competency development, thus reducing their willingness to leave.

#### Organizational level

**FIGURE 1:**
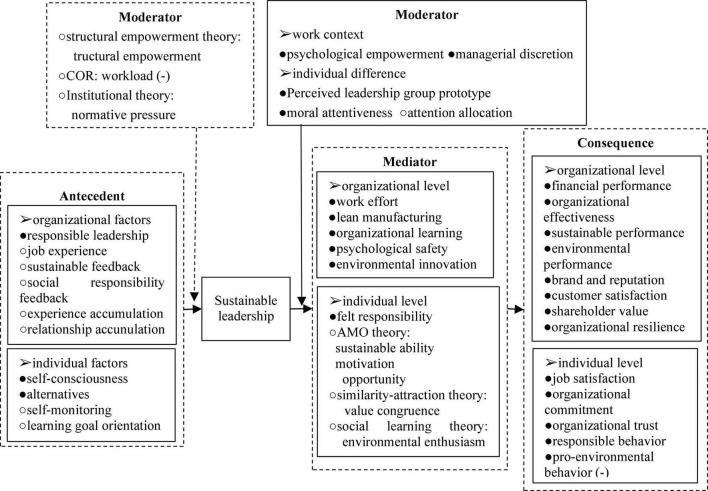
The analysis framework for sustainable leadership. Solid arrows and “•” represent pathway relationships and variables explored by existing studies, dashed arrows and “°” represent pathway relationships and variables proposed by research outlooks, and (−) indicates negative correlations.

At the organizational level, the consequences of sustainable leadership are mainly reflected in performance-related variables, and the relationship of them has received more attention from scholars. For example, Avery and Bergsteiner (2011b) stated that sustainable leadership produced 5 performance-related outcomes, namely reputation, customer satisfaction, finances, shareholder value, and long-term value for multiple stakeholders. They also pointed to the ability of sustainable leadership to improve organizational resilience. An empirical analysis by Suriyankietkaew and Avery (2016) with a sample of Thai SMEs confirmed a significant positive relationship between 16 of the 23 sustainable leadership practices and corporate financial performance. Studies by Lee (2017), Sezgin-nartgun et al. (2020) also pointed out that sustainable leadership can enhance organizational effectiveness. Recently, empirical analyses by scholars have mainly linked the outcomes of sustainable leadership to organizational sustainability. Burawat (2019) and Iqbal et al. (2020a,b) examined the effects of sustainable leadership in numerous SMEs in different countries, and found that sustainable leadership had a positive impact on sustainable performance. In addition, Fatoki’s (2021) analysis with a sample of hotel companies confirmed the positive relationship between sustainable leadership and sustainable performance. Empirical studies by Javed et al. (2020) and Iqbal et al. (2020a) based on many SMEs in Asian coastal countries had shown that sustainable leadership had a significant positive effect on environmental performance. Moreover, scholars have further explained the mechanism of the effect of sustainable leadership on performance-related consequences through mediating variables and moderating variables. Based on the above research, we develop a research framework for sustainable leadership (shown in Figure 1). And the research framework also contains related content in the research prospects.

## Research prospects

### Deepening the conception of sustainable leadership

Although the number of sustainable leadership research is limited, scholars still differ greatly in their understanding of its connotation, dimensional delineation, and measurement, and have yet to reach a consensus, which has seriously hindered the further development of sustainable leadership. In terms of connotation and dimensional division, most scholars agree that sustainable leadership is multidimensional (Gerard et al., 2017), but there are many overlaps of the dimensional division between sustainable leadership and transformational leadership, ethical leadership, and responsible leadership in existing studies, resulting in numerous questions on sustainable leadership dimensions and measurement questionnaires, which not only affects the theoretical development of sustainable leadership, but also limits the related empirical analysis. Therefore, future research can further clarify the unique structure of sustainable leadership, identify its similarities and differences with other leadership styles, and develop a practical measurement questionnaires based on this.

### Enriching the antecedents of sustainable leadership

Future research could explore its potential antecedent variables from a dynamic perspective. The focus of research on sustainable leadership ignores the fact that sustainable leadership behavior may change or fluctuate over time, and thus tapping into its antecedent variables at only one point in time clearly fails to capture changes in sustainable leadership behaviors. Based on existing research, the dynamics of sustainable leadership behavior can be of two kinds, namely, transformation and growth of leadership behavior (McClean et al., 2019). In terms of the study of antecedent variables in the perspective of sustainable leadership transformation, we hypothesize that the main factors that trigger sustainable leadership include experiences, interactions, and conditional triggers. Experiential triggers refer to discrete, work-related experiences of the leader that may cause the leader to face new challenges in the workplace and thus trigger a change in the leader’s behavior; interactive triggers refer to interactive factors that can change the leader’s behavior, such as feedback. Sustainability feedback, social responsibility feedback, etc. may improve sustainable leadership; conditional triggers refer to triggers that are conditional on other factors, such as specific conditional events. In terms of antecedent variables from a sustainable leadership growth perspective, we hypothesize that the triggers for sustainable leadership include experience, relationship building, and traits and orientations. Because sustainable leadership requires leaders to have a broad focus, not only on developing people and teams and improving organizational operations, but also on social wellbeing, socially responsible outputs, and environmental protection, the formation of sustainable leaders is hardly abrupt, but rather evolves over time, with experience leading to higher levels of skills and competencies, and the participatory behaviors of more experienced and established leaders being more effective, making it more likely that the accumulation of experience will shape high-level sustainable leaders over time. In addition, certain traits of leaders, such as self-monitoring, self-efficacy, boldness, commitment, and charisma, may inspire sustainable leadership.

### Uncovering the moderators of sustainable leadership

We speculate on the possible weighting factors affecting sustainable leadership formation from the perspectives of structural empowerment theory, resource conservation theory, and institutional theory. First, structural empowerment theory states that power sharing, such as the granting of rights and the delegation of tasks, can enhance individual engagement (Wang et al., 2022). Since sustainable leadership is a rich leadership behavior that requires leaders to balance economic, social, and environmental aspects, it means that leaders need to have a lot of information and resources, and even continuous development opportunities, etc. In a high structural empowerment work environment, leaders are more likely to have access to rich information, support, and resources, thus creating a good working environment for their sustainable leadership behavior. Second, according to resource conservation theory, leaders have limited resources available to them, and the resources they consume to engage in an activity affect their resource investment in other activities. When the workload at work is high, overload reduces the likelihood that leaders will exhibit sustainable leadership behaviors because their need to handle high loads reduces leaders’ perceived progress toward their goals and also causes them to prioritize economic tasks, which leads them to reduce sustainable leadership behaviors. Finally, as open systems, managers’ leadership behaviors are also influenced by external contextual factors. In social contexts, managers’ perceived external normative pressures influence their sustainable leadership behaviors, and in order to gain sustained social support and legitimacy, managers will act to respond to public expectations in a timely manner and position themselves as key contributors to social sustainability, thereby assuming greater social and environmental responsibility. Therefore, future research could also explore the moderating effects that normative pressures play in the formation of sustainable leaders based on institutional theory.

### Exploring the mediators of sustainable leadership

While studies on the mechanisms of sustainable leadership have focused on the organizational level, this paper seeks to suggest possible mediating variables between sustainable leadership and its outcomes at the individual level with the help of relevant theories. Specifically, this paper applies AMO theory, similar attraction theory, and social learning theory to propose corresponding perspectives. First, AMO theory states that employees’ behavior or performance is determined by their abilities, motivation and opportunities, and that leaders’ leadership behaviors can have an impact on employees’ abilities, motivation and opportunities. Based on this, sustainable leadership is used as a starting point to explore the mechanisms underlying the influence of sustainable leadership on employees’ sustainability behaviors, in which AMO factors necessarily play a mediating role. First, the inherent explanatory mechanism centered on competencies. In sustainable activities, employees need to possess certain sustainable competencies, i.e., the mental and cognitive abilities of employees related to effectively engaging in a sustainable activity, including knowledge and skills related to sustainable activities, etc. Sustainable leaders enhance the sustainability of their employees by setting sustainable role models for them in their daily management process, and they also provide the necessary resources to enhance sustainability, such as enhanced coaching and training, and the creation of a positive environment. Second, with motivation as the core explanatory mechanism, internal motivation is the most favorable motivating factor that drives employees to engage in an activity. In sustainable activities, internal motivation that can inspire sustainable behaviors carries the same sustainability, reflecting sustainable behaviors implemented by employees out of their love for sustainable activities. Sustainability leadership uses organizational culture to promote sustainability concepts, values, and goals within the organization to induce internal motivation for sustainability. Third, opportunity-centered explanatory mechanisms, in general, refer to factors in the work environment that can promote individual behavior, such as organizational policies and working conditions. In sustainability activities, the opportunities that can drive employee behavior are primarily sustainability opportunities, i.e., a set of policies, conditions, elements, etc. that are conducive to improving employee sustainability behavior. Sustainable leadership can provide opportunities for employees to implement sustainable behaviors, such as providing relevant training and development opportunities and shaping a sustainable organizational culture.

## Author contributions

YL contributed to conducting the literature review, designing the research, collecting some of the data, analyzing the data, drafting the manuscript, meanwhile, repeatedly revised, and refined the content of the manuscript.
